# Neurophobia and its implications: evidence from a Caribbean medical school

**DOI:** 10.1186/1472-6920-9-39

**Published:** 2009-07-01

**Authors:** Farid F Youssef

**Affiliations:** 1 Department of Preclinical Sciences, The University of the West Indies, St Augustine Campus, St Augustine, Trinidad & Tobago

## Abstract

**Background:**

Neurology is regarded as a difficult component of the medical curriculum. This has been so marked that the term neurophobia and its effects are being investigated. Given the impact of neurological disorders worldwide, neurophobia has the potential to affect the diagnosis and management of such cases.

**Methods:**

A cross-sectional survey was done among clinical fourth and fifth year students at the Faculty of Medical Sciences, University of the West Indies, St. Augustine, Trinidad & Tobago. A survey tool successfully used in other schools was adapted to assess perceived level of difficulty, knowledge and interest in various medical sub-specialties including, neurology, cardiology, psychiatry, geriatrics, endocrinology, respiratory medicine, gastroenterology and pediatrics. Questions asked included: *"What is your current level of interest in the following medical specialties?"; "What is your current level of knowledge in the given medical specialties?"; "Do you think the subject is easy or difficult?" and "Why do you think neurology is difficult?" *Students were required to answer using a Likert scale and results were tabulated into mean scores and standard errors.

**Results:**

The response rate was 65% (167/255). Neurology was identified as the subject which students found most difficult (score 3.89 ± 0.068) and had least knowledge of (2.32 ± 0.075). These scores were significantly different from those observed for the other disciplines (p < 0.001). The need to know basic neuroscience was identified as the biggest contributor to the difficulty associated with neurology (3.89 ± 0.072) followed closely by the complex clinical examination associated with neurology (3.69 ± 0.072). Greater clinical and practical exposure, more time being spent on the subject, and improved teaching skills of lecturers were put forward as suggestions for improving the teaching of neurology.

**Conclusion:**

This study provides empirical evidence that 'neurophobia' may indeed exist among the student population of the school. It suggests the need to re-visit the approach to neuroscience and neurology education and is consistent with similar trends worldwide.

## Background

Historically, neurology and neurosciences in general have been regarded as one of the more difficult components of the traditional medical curriculum. While such perceptions were anecdotal for many years, the last 10–15 years have seen attempts to test and describe these assertions.

In 1994 the term neurophobia was coined by Jozefowicz to describe *'the fear of neural sciences and neurology' *among medical students and even doctors [1]. While Jozefowicz did not present scientific data to support his claim, evidence for neurophobia has now been reported from different sources. Most recently it was noted that among medical students and junior doctors in Ireland neurology was perceived as the most difficult of eight medical sub-specialties assessed [2]. Similar data was observed in a 2002 report that reviewed medical students from two London medical schools, general practitioners and senior house officers. This latter study concluded that neurology was perceived as the most difficult of the sub-specialties, students felt they were least knowledgeable about this subject and doctors had least confidence in themselves when managing neurological cases [3].

The reasons for neurophobia are not clear. It has been suggested that the manner in which neuroscience and neurology are taught may be the cause. Another UK study seeking to explore this observed no evidence of neurophobia when: (i) a modified delivery platform that focused upon increasing the length of time spent on neurology was used and (ii) focusing of the course deliverables took place [4]. Other factors responsible for neurophobia may include the complex subject matter, its sometimes abstract nature, the length of time that must be devoted in order to elicit clinical signs and possibly the fact that neurologists themselves may enjoy the perceived notion that theirs is a difficult subject only suited for the most brilliant [3,5].

Neurophobia, if it does exist, has profound implications for the practice of health care in any nation. The World Health Organization (WHO) estimates that neurological conditions contribute approximately 6.3% to the global health burden [6] and are responsible for 12% of global mortality. In Britain it was estimated that about 10% of all persons presenting to general practioners (GP) had a neurological complaint [7] and 28% of disability among the general population is secondary to neurological or psychiatric problems. Thus there is a significant burden of neurological disease globally and this may be exaggerated among populations in certain parts of the world, like the Caribbean, where a number of neurological diseases, including epilepsy and psychiatric disorders, are misunderstood and even subject to stigmatization.

Another consequence of neurophobia may be its impact on the number of persons who chose to specialize in neurology and other neuroscience based sub-specialties. In the UK there are less neurologists practising than in other disciplines and though hard evidence is lacking in the Caribbean, a review of the yellow pages in Trinidad & Tobago tends to suggest this may also be the case. Interestingly a recent report from Trinidad & Tobago observed that first year medical students are least inclined to chose psychiatry as their preferred specialization [8].

Given the evidence of neurophobia from other medical schools, and its implication for public health, we have sought to assess the extent of neurophobia among the clinical medical students at our school, their perceptions towards neurology and suggestions for improvements in teaching neurology and neuroscience.

## Methods

This study was approved by the Faculty Ethics Committee. It was designed as a cross-sectional study to assess neurophobia among fulltime fourth and fifth year medical students of the Faculty of Medical Sciences, The University of the West Indies at the St. Augustine Campus, Trinidad & Tobago.

A questionnaire was designed based upon that developed and successfully used in other studies [2,3]. The questionnaire collected minimal epidemiological data including age and gender before asking a series of questions to assess difficulty, interest and knowledge among differing medical sub-specialties. Key questions were:

• What is your current level of interest in the following medical specialties? Scored on 6 pt scale: 0 = not known/other; 1 = little or no interest; 2 = some interest; 3 = moderate interest; 4 = quite interested; 5 = very interested

• What is your current level of knowledge in the given medical specialties? Scored on a 6 pt scale: 0 = not known or other; 1 = little or none; 2 = some; 3 = moderate; 4 = fair; 5 = great.

• Do you think the subject is easy or difficult? Scored on a 6 pt scale: 0 = not known or other; 1 = very easy; 2 = quite easy; 3 = moderate; 4 = quite difficult; 5 = very difficult.

Eight specialties were included in the original questionnaire but geriatrics was subsequently excluded from the analysis due to the fact that it is not focused upon as a sub-discipline at any point during the training of our medical students. The final part of the instrument focused on reasons why neurology may be perceived as difficult and ways in which improvements can be made. Questions were based upon the results of Schon *et al *(2002) and students were also asked to assess how the following factors contributed to the difficult nature of neurology: (i) The need to know basic neuroscience; (ii) The complex clinical examination; (iii) Neurology having a reputation as a difficult subject; (iv) Neurology covering such a large number of diagnoses and (v) Neurology being badly taught.

Questionnaires were distributed to students in their clinical clerkship groups and students were then allowed to complete the questionnaire off site. Completed questionnaires were then collected at a later date. In all 167 completed questionnaires were collected from 90 fourth year students and 77 fifth year students. We cannot guarantee that every registered student received a questionnaire but using total enrollment as our total population the response rate for fourth year was 90/124 (73%) and for fifth year 77/131 (59%). Total response rate was 65%.

### Statistical analysis

Data was analyzed using the SPSS Version 12.0. Means and standard errors were calculated for questions where applicable and comparison of means done using a Student's t-test. The α-error was set at p < 0.05.

## Results

**Figure 1:**
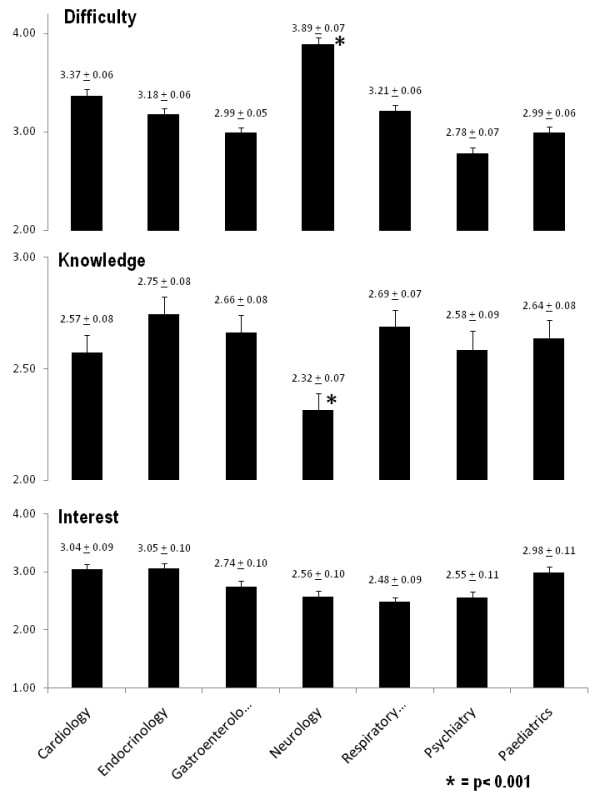
**Histograms demonstrating the mean scores plus standard errors for three questions in relation to difficulty with, current knowledge of, and level of interest in selected medical sub-specialties**.

When students were asked to assess the difficulty of various medical sub-specialties neurology was rated as the most difficult with a mean score of 3.89 ± 0.068. This result was statistically significant (p < 0.001) and 0.52 points higher than the subject considered second most difficult, cardiology 3.37 ± 0.063 (Figure 1). Twenty-four percent (24%) of respondents identified neurology as being 'very difficult', much higher than those identified for any other subject, cardiology again being ranked second at 9%. All other specialties had less than 5% of students identifying it as 'very difficult'.

When students were asked to rate their perceived knowledge of sub-specialties, neurology received the lowest mean score, 2.32 ± 0.075, (p < 0.001). Cardiology and psychiatry were ranked second and third respectively (Figure 1). Neurology however was not the subject which students had least interest in, average score 2.56 ± 0.01 ranking 5th ahead of respiratory medicine 2.48 ± 0.088 and psychiatry 2.55 ± 0.106. There was no significant difference between the level of interest among the sub-specialties assessed (p > 0.05). Neurology did have the second highest percentage of students (26%) indicating very little interest in the subject, behind psychiatry (29%).

**Figure 2:**
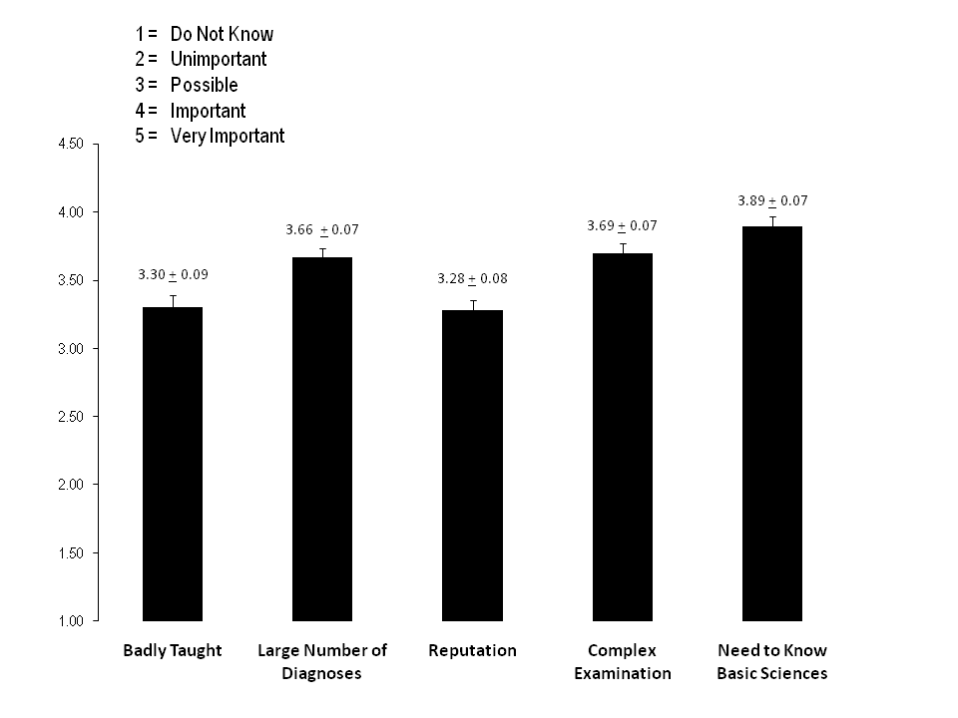
**Histograms demonstrating mean scores plus standard errors when students were asked to assess reasons for difficulty with neurology**.

Students were asked to rate why they thought neurology was difficult and these results are summarized in figure 2. The need to know basic neuroscience and the complex clinical examination were identified as the two factors that had the greatest impact with means of 3.89 ± 0.072 and 3.69 ± 0.072 respectively. These were the only two factors that also had a mode of 4 (important).

Students were also asked the open ended question, to describe ways in which adjusting the teaching of neuroscience and neurology could help improve the situation. Of the 167 responses a large proportion of students did not answer this question or indicated that the teaching as is, was adequate (73 persons in total). Other responses clustered around 4 areas. The first was building more clinical or practical exposure into the teaching (39 persons; 23%). Twenty two students (13%) felt more time was necessary to be devoted to the teaching of the subject, and 16 students (10%) felt there was a need for improved teaching tools, in particular audio-visual equipment. Finally 27 students (16%) identified the need for the actual lecturing skills of the teachers to be improved or teaching needing to be better organized.

## Discussion

Neurology has traditionally been perceived as one of the more difficult clinical sub-specialties and as such the term neurophobia was coined to describe *'the fear of neural sciences and neurology among medical students and doctors' *[1]. Though originally unsubstantiated by scientific data, the last few years has brought a renewed focus upon the teaching of neurology and more empirical studies seeking to address this issue [9-11].

Our medical school was established in 1989 and currently has an intake of approximately 200 medical students annually. Its curriculum has followed a traditional British model with a preclinical phase lasting 3 years and a clinical phase lasting 2 years before graduation and internship. Since its inception it has inculcated Problem Based Learning (PBL) as a core teaching methodology, especially during the preclinical years, designed to give students early introduction to clinical scenarios and improve their critical thinking and decision making skills. This was hoped to bear fruit in the clinical years and one recent study notes that it has at least 'broken even' [12].

We report that despite a PBL approach in the pre-clinical years our students still ranked neurology as the most difficult of seven medical sub-specialties by a fairly substantial margin, with approximately one quarter of all students indicating they found neurology very difficult. Similar results have been reported in other studies out of the United Kingdom and Ireland [2,3]. Interestingly it was suggested that increasing the PBL component of the curriculum could be one means of helping to address this situation but our results indicate that PBL by itself will not adequately address the situation.

These results were borne out when students were asked to assess the subject that they felt they had least knowledge in and neurology again scored unfavorably, having the lowest mean. Coupled with the perceived difficulty associated with neurology these findings may have significant implications. Schon *et al *(2002) were able to extend their study to include junior doctors and it was noted that they had least confidence when dealing with neurological cases 'at the bedside' [3]. This suggests that attitudes and perceptions developed in medical school may have the tendency to spill over into clinical practice. Alternatively doctors' lack of confidence may be the result of inadequate training that if remedied would greatly improve performance on graduation. Whatever the case the onus is upon the academic staff to address these issues and ensure that students feel more empowered to deal with neurological cases.

Ridsdale *et al *(2007) examined the possible effects of neurophobia in clinical practice within the United Kingdom. They noted that neurological diseases are increasing among the general population and this coupled with a lack of confidence among general practioners often results in over referrals to specialists [4]. At the same time, the UK had the lowest number of neurologists per captia in Europe, an issue that is also of some concern in the Caribbean. In essence we may have a dilemma where neurophobia creates fewer specialists but then also places more demand on the few that do exist.

The onus therefore must be on improved training of our medical graduates. In fact we observed that neurology was not recorded as the discipline in which students had least interest coming ahead of respiratory medicine and psychiatry (though it is worth noting that the latter is a neuroscience based discipline) a finding similar to other studies [3]. When students were themselves asked to indicate why neurology proved to be so difficult two factors stood out: the need to know basic neuroscience and the complex clinical examination. These were underscored by the responses to a similar open ended question in which students requested more time for neuroscience and neurology teaching and more clinical/practical exposure.

Within our context basic neuroscience is taught as an eight week course in the second year of medical school. Though heavily drawing on PBL it seems to suggest that this not enough time for the students to assimilate the material required. Neurology is taught in the clinical years but not as an independent clerkship; rather it is bundled inside of the larger medicine clerkship. This arrangement for teaching neuroscience and neurology may lack focus and based on our results does not allow enough time for assimilation of the material taught.

Data from the recently formed GKT Medical School in London (the largest medical school in the United Kingdom) suggest that if more time is allocated to teaching of neurology and neuroscience and this is coupled to focused course objectives, neurophobia is reduced. Students though, still perceived neurology as a difficult subject and lacked confidence in approaching problems [4]. Other approaches to improve neurology teaching using case-based teaching in Australia [13] and teaching videos in Singapore [14] have also met with some success.

These interventions are clearly not complete solutions in themselves but represent movement in the right direction. They suggest that modifications in curriculum and teaching methodology can have a positive effect upon learning and such practices should be considered elsewhere. They also highlight that in our context, though PBL has brought with it some success, there is still a need to evaluate and bring refinements consistent with latest pedagogical data. Ultimately though, neurology as a sub-specialty may indeed be more difficult than other subjects and teachers must simply take the time and use all resources available to ensure adequate learning takes place.

Finally it may be worthwhile considering the perception students have of various subjects upon entering medical school. A recent study in the Caribbean noted a definite bias against psychiatry [8]. In a similar manner perceptions of neurology may have nothing to do with teaching or curriculum but may be the results of societal stereotypes that need to be addressed in a more broad sense as a part of the overall solution. A British Medical Journal (BMJ) editorial describes *"the neurologist is one of the great archetypes: a brilliant, forgetful man with a bulging cranium....who....talks with ease about bits of the brain you'd forgotten existed, adores diagnosis and rare syndromes, and – most importantly – never bothers about treatment." *Schon *et al *even suggests that such a reputation is possibly enjoyed and encouraged by neurologists who like the notion that neurology is a discipline for which only *'young Einsteins need apply' *[3]. Such stereotypes clearly are not consistent with the future of neuroscience and neurology and the emerging clinical demands of the 21^st ^century.

### Future Directions

This study highlights that neurophobia is indeed a problem among our students but having identified the problem solutions need to be considered. The students themselves have highlighted the need for increased clinical exposure and this must be considered. The Medical School in Mona, Jamaica has introduced bedside teaching from year one and it would be interesting to compare attitudes to neurology among those students as compared to ours. In addition given the general feeling that basic neuroscience is difficult, it is easy to suggest that more time be allocated to the subject. However, this is not readily achievable and is also the desire of almost all other disciplines. The solution perhaps lies in identifying topic areas that have most relevance to doctors in training and streamlining the curriculum; efforts along these lines are in fact in train across the faculty. Along these lines there is also a push to increase vertical integration throughout the curriculum which would increase clinical exposure in the early years of training and also allow the revisiting of basic science concepts during the clinical years.

### Limitations

One obvious limitation of this study was the response rate of 65%. During the clinical years our students function in small group clerkships and rarely come together as a whole group. Given this limitation we distributed the questionnaires within the clerkships and allowed the students to complete them off site. Greater efforts could have been made to follow up with individual students to ensure a higher response rate but this was limited by manpower and the students being spread across four hospitals in different parts of the country. This meant that only the more motivated and perhaps more conscientious students returned the questionnaires, hence the response rate of sixty five percent. However it is to be expected that this population would probably also have been more focused in their attempts to 'come to grips' with neurology and so an increased response rate may have been expected to further highlight the problem of neurophobia.

## Conclusion

In conclusion we have demonstrated that neurophobia does indeed exist among our student population. While the use of PBL is still to be encouraged, the same principles that underlie its success need to be fully embraced throughout the entire medical curriculum and combined with the latest findings in medical education, to create a more focused and practical approach to neuroscience/neurology education. We suggest these results add to the growing body of data that highlights an increasing awareness for the need to modify neuroscience and neurology training to better meet the needs of the general population.

## Competing interests

The author declares that they have no competing interests.

## Authors' contributions

FFY was responsible for the conceptualization, coordination, design and statistical analysis of this study. FFY was responsible for writing the manuscript.

## Pre-publication history

The pre-publication history for this paper can be accessed here:


